# 3-(2-Eth­oxy­phen­yl)-1-(3-nitro­phen­yl)triaz-1-ene

**DOI:** 10.1107/S1600536811048781

**Published:** 2011-11-30

**Authors:** Mohammad Reza Melardi, Atefeh Ghannadan, Mitra Peyman, Giuseppe Bruno, Hadi Amiri Rudbari

**Affiliations:** a Department of Chemistry, Islamic Azad University, Karaj Branch, Karaj, Iran; b Dipartimento di Chimica Inorganica, Universita di Messina, Messina, Italy

## Abstract

The title compound, C_14_H_14_N_4_O_3_, exhibits a *trans* geometry about the N=N double bond in the triazene unit. The mol­ecule is approximately planar (r.m.s. deviation = 0.044 Å for all non-H atoms). An intra­molecular N—H⋯O hydrogen bond occurs. In the crystal, C—H⋯N hydrogen bonds lead to the formation of dimers which are, in turn, connected to each other by C—H⋯O hydrogen bonds, forming infinite chains of *R*_2_^2^(8) graph-set motif.

## Related literature

For aryl triazenes, their structural properties and metal complexes see: Meldola & Streatfield (1888 ▶); Leman *et al.* (1993 ▶); Chen *et al.* (2002 ▶); Vrieze & Van Koten (1987 ▶). For a similar structure with cyano instead of eth­oxy groups, see: Melardi *et al.* (2008 ▶). For the synthesis and characterization of a similar structure with meth­oxy instead of eth­oxy groups, see: Rofouei *et al.* (2006 ▶). For the synthesis and crystal structures of mercury(II) and silver(I) complexes with 1,3-bis­(2-meth­oxy­phen­yl)triazene, see: Hematyar & Rofouei (2008 ▶) and Payehghadr *et al.* (2007 ▶), respectively. For hydrogen-bond patterns and related graph sets, see: Grell *et al.* (2002 ▶). 
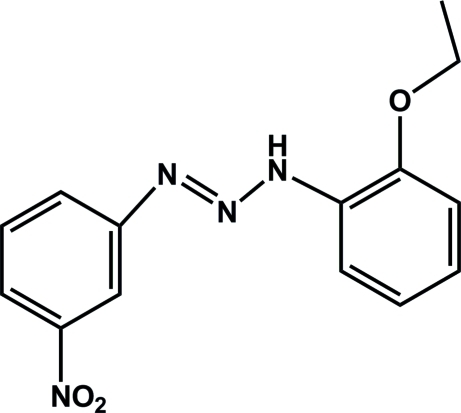


## Experimental

### 

#### Crystal data

C_14_H_14_N_4_O_3_
*M* *_r_* = 286.29
Triclinic, 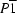

*a* = 6.7754 (4) Å
*b* = 7.5482 (4) Å
*c* = 14.0467 (7) Å
α = 99.057 (3)°
β = 102.479 (2)°
γ = 90.192 (3)°
*V* = 692.14 (6) Å^3^
*Z* = 2
Mo *K*α radiation
μ = 0.10 mm^−1^
*T* = 293 K
0.55 × 0.33 × 0.26 mm

#### Data collection

Bruker APEXII CCD diffractometer
Absorption correction: multi-scan (*SADABS*; Sheldrick, 2004 ▶) *T*_min_ = 0.688, *T*_max_ = 0.746
26097 measured reflections
3178 independent reflections
2693 reflections with *I* > 2σ(*I*)
*R*_int_ = 0.019

#### Refinement

*R*[*F* ^2^ > 2σ(*F* ^2^)] = 0.039
*wR*(*F* ^2^) = 0.115
*S* = 1.06
3178 reflections
191 parameters
H-atom parameters constrained
Δρ_max_ = 0.19 e Å^−3^
Δρ_min_ = −0.17 e Å^−3^

### 

Data collection: *APEX2* (Bruker, 2005 ▶); cell refinement: *SAINT-Plus* (Bruker, 2005 ▶); data reduction: *SAINT-Plus*; program(s) used to solve structure: *SHELXS97* (Sheldrick, 2008 ▶); program(s) used to refine structure: *SHELXL97* (Sheldrick, 2008 ▶); molecular graphics: *SHELXTL* (Sheldrick, 2008 ▶); software used to prepare material for publication: *SHELXTL*.

## Supplementary Material

Crystal structure: contains datablock(s) I, global. DOI: 10.1107/S1600536811048781/qm2042sup1.cif

Structure factors: contains datablock(s) I. DOI: 10.1107/S1600536811048781/qm2042Isup2.hkl

Supplementary material file. DOI: 10.1107/S1600536811048781/qm2042Isup3.cml

Additional supplementary materials:  crystallographic information; 3D view; checkCIF report

## Figures and Tables

**Table 1:** Hydrogen-bond geometry (Å, °)

*D*—H⋯*A*	*D*—H	H⋯*A*	*D*⋯*A*	*D*—H⋯*A*
N1—H1⋯O1	0.86	2.26	2.6130 (12)	105
C7—H2*A*⋯O3^i^	0.97	2.55	3.4595 (18)	157
C10—H10⋯N3^ii^	0.93	2.65	3.543 (3)	161

Symmetry codes: (i) 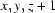; (ii) 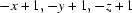.

**Fig. 1.**
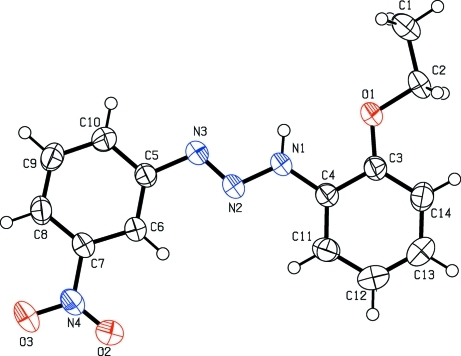
Molecular structure of the title compound. Thermal ellipsoids are drawn at the 50% probability level.

**Fig. 2.**
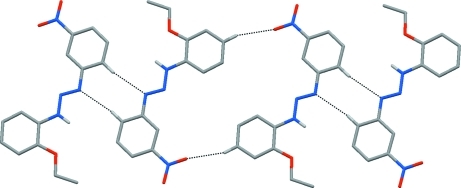
C—H···N and C—H···O hydrogen bonds connect the different units into chains with R22(8) graph-set motifs

**Fig. 3.**
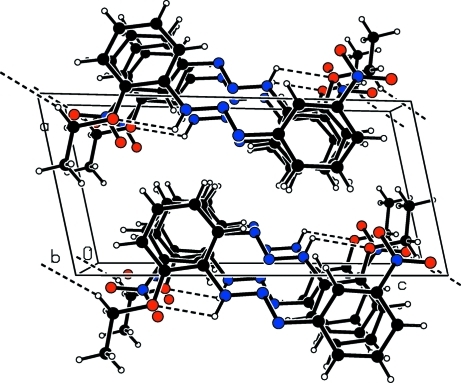
Unit cell packing diagram of the title compound, hydrogen bonding are shown as dashed lines.

## supplementary crystallographic information

### Comment

Aryl triazenes have been studied for over 130 years for their interesting structural, anticancer, and reactivity properties. The first extensive investigation of the coordination chemistry of a triazene derivative (1,3 diphenyltriazene) was carried out in 1887 by Meldola (Meldola *et al.*, 1888). In the intervening years, numerous transition metal triazenide compounds have been studied (Leman *et al.*, 1993). Triazene compounds characterized by having a diazoamino group commonly adopt a *trans* configuration in the ground state (Chen *et al.*, 2002). The study of transition metal complexes containing 1,3-diaryltriazenide [RN═N—NR]- ligands has increased greatly in the past few years, because of their potential reactivity in relation to their several coordination modes (Vrieze *et al.*, 1987). We have recently reported the synthesis and characterization of the two molecules 1,3-bis(2-methoxyphenyl)triazene (Rofouei, *et al.*, 2006) and 1,3-bis(2-cyanophenyl)triazene (Melardi, *et al.*, 2008).

The title compound, C_14_H_14_N_4_O_3_, is a related triazene compound. It exhibits a *trans* stereo chemistry of the N═N double bond, and the C9—N3—N2—N1 and C1—N1—N2—N3 torsion angles are -179.23 (9) and 177.91 (10)°, respectively which indicates the molecule is planar. The N1—N2 and N2—N3 bond distances are 1.3295 (13) and 1.2550 (14) Å, respectively, which indicates the presence of distinct single and double bonds between the nitrogen atoms. These values are in good agreement with the reported data for N—N and N═N bond distances (Hematyar, *et al.*, 2008; Payehghadr, *et al.* 2007). For example, in 1,3-bis(2-cyanophenyl)triazene, the N—N and N═N bond distances are 1.335 (5) and 1.289 (5) Å (Melardi, *et al.*, 2008). Individual molecules are mostly planar with an r.m.s. deviation from planarity of 0.044 Å for all non-hydrogen atoms. Every molecule in the molecular structure (Fig. 1) is connected to other unit by two distinct C—H···N hydrogen bonds to form dimers. The resultant dimers are then connected to each other by C—H···O hydrogen bonds to form infinite chains with *R*^2^_2_(8) graph-set motifs (Grell *et al.*, 2002)) (Fig. 2). Unit cell diagram of the title compound is illustrated in Fig. 3.

### Experimental

The compound was prepared by the following method: A 100 ml flask was charged with 10 g of ice and 15 ml of water and then cooled to 273 k in an ice-bath. To this was added 2 mmol (0.344 g) of 3-nitroaniline and 2 mmol of hydrochloric acid (36.5%) and 2 ml of water. To thissolution was then added a solution containing NaNO_2_ (2 mmol, 0.16 g) in 2 ml of water during a 15 min period. After mixing for 15 min, the obtained solution was added to a solution of 2 mmol (0.261 ml) of *o*-phenetidin and 2 ml of methanol and 2 ml of water.

After that a solution containing 36 mmol (2.95 g) of sodium acetate in 10 ml of water was added. After mixing for 24 h the orange product was filtered off and dissolved in DMSO. Recrystallization from DMSO afforded the product as an orange crystalline material. ^1^H NMR (300MHZ, DMSO): 1.37(6*H*, CH_3_), 4.12(4*H*, CH_2_), 6.98–8.07 (8*H*, aromatic), 12.93(1*H*, NH). IR (KBr): 3326, 1484, 1468, 1253, 1046, 816 cm^-1^

### Crystal data

**Table:**

C_14_H_14_N_4_O_3_	*Z* = 2
*M**_r_* = 286.29	*F*(000) = 300
Triclinic, *P*1	*D*_x_ = 1.374 Mg m^−^^3^
Hall symbol: -P 1	Mo *K*α radiation, λ = 0.71073 Å
*a* = 6.7754 (4) Å	Cell parameters from 9946 reflections
*b* = 7.5482 (4) Å	θ = 2.7–31.1°
*c* = 14.0467 (7) Å	µ = 0.10 mm^−^^1^
α = 99.057 (3)°	*T* = 293 K
β = 102.479 (2)°	Irregular, colourless
γ = 90.192 (3)°	0.55 × 0.33 × 0.26 mm
*V* = 692.14 (6) Å^3^	

### Data collection

**Table:**

Bruker APEXII CCD diffractometer	3178 independent reflections
Radiation source: fine-focus sealed tube	2693 reflections with *I* > 2σ(*I*)
graphite	*R*_int_ = 0.019
φ and ω scans	θ_max_ = 27.5°, θ_min_ = 2.7°
Absorption correction: multi-scan (*SADABS*; Sheldrick, 2004)	*h* = −8→8
*T*_min_ = 0.688, *T*_max_ = 0.746	*k* = −9→9
26097 measured reflections	*l* = −18→18

### Refinement

**Table:**

Refinement on *F*^2^	Primary atom site location: structure-invariant direct methods
Least-squares matrix: full	Secondary atom site location: difference Fourier map
*R*[*F*^2^ > 2σ(*F*^2^)] = 0.039	Hydrogen site location: inferred from neighbouring sites
*wR*(*F*^2^) = 0.115	H-atom parameters constrained
*S* = 1.06	*w* = 1/[σ^2^(*F*_o_^2^) + (0.0511*P*)^2^ + 0.145*P*] where *P* = (*F*_o_^2^ + 2*F*_c_^2^)/3
3178 reflections	(Δ/σ)_max_ < 0.001
191 parameters	Δρ_max_ = 0.19 e Å^−^^3^
0 restraints	Δρ_min_ = −0.17 e Å^−^^3^

### Special details

**Table:**

Geometry. All s.u.'s (except the s.u. in the dihedral angle between two l.s. planes) are estimated using the full covariance matrix. The cell s.u.'s are taken into account individually in the estimation of s.u.'s in distances, angles and torsion angles; correlations between s.u.'s in cell parameters are only used when they are defined by crystal symmetry. An approximate (isotropic) treatment of cell s.u.'s is used for estimating s.u.'s involving l.s. planes.
Refinement. Refinement of *F*^2^ against ALL reflections. The weighted *R*-factor *wR* and goodness of fit *S* are based on *F*^2^, conventional *R*-factors *R* are based on *F*, with *F* set to zero for negative *F*^2^. The threshold expression of *F*^2^ > 2σ(*F*^2^) is used only for calculating *R*-factors(gt) *etc*. and is not relevant to the choice of reflections for refinement. *R*-factors based on *F*^2^ are statistically about twice as large as those based on *F*, and *R*- factors based on ALL data will be even larger.

### Fractional atomic coordinates and isotropic or equivalent isotropic displacement parameters (Å^2^)

**Table:**

	*x*	*y*	*z*	*U*_iso_*/*U*_eq_	
O1	0.14363 (13)	0.78956 (13)	0.80767 (6)	0.0496 (2)	
O2	−0.25541 (16)	0.75287 (19)	0.17852 (8)	0.0775 (4)	
O3	−0.09609 (19)	0.67215 (19)	0.06405 (7)	0.0772 (4)	
N1	0.08879 (15)	0.75248 (15)	0.61555 (7)	0.0463 (3)	
H1	0.1969	0.7224	0.6532	0.056*	
N2	0.07393 (15)	0.72890 (13)	0.51841 (7)	0.0405 (2)	
N3	0.22616 (15)	0.65698 (14)	0.49393 (7)	0.0452 (2)	
N4	−0.10788 (17)	0.69283 (16)	0.15046 (7)	0.0510 (3)	
C8	0.3938 (2)	0.7498 (2)	0.94857 (10)	0.0657 (4)	
H1A	0.3911	0.6227	0.9261	0.099*	
H1B	0.4296	0.7732	1.0195	0.099*	
H1C	0.4919	0.8082	0.9226	0.099*	
C7	0.1898 (2)	0.8201 (2)	0.91353 (9)	0.0539 (3)	
H2A	0.0890	0.7591	0.9376	0.065*	
H2B	0.1895	0.9476	0.9383	0.065*	
C2	−0.03776 (17)	0.84559 (16)	0.76042 (8)	0.0408 (3)	
C1	−0.06778 (17)	0.82518 (15)	0.65747 (8)	0.0388 (2)	
C9	0.21578 (17)	0.63226 (15)	0.39086 (8)	0.0383 (2)	
C14	0.05175 (17)	0.67526 (15)	0.32130 (8)	0.0385 (2)	
H6	−0.0627	0.7236	0.3402	0.046*	
C13	0.06377 (18)	0.64406 (15)	0.22357 (8)	0.0404 (3)	
C12	0.2299 (2)	0.57199 (18)	0.19153 (9)	0.0488 (3)	
H8	0.2329	0.5523	0.1248	0.059*	
C11	0.3909 (2)	0.53033 (18)	0.26185 (10)	0.0529 (3)	
H9	0.5049	0.4818	0.2425	0.063*	
C10	0.38486 (19)	0.55994 (17)	0.36067 (9)	0.0466 (3)	
H10	0.4946	0.5313	0.4074	0.056*	
C6	−0.24781 (19)	0.87423 (17)	0.60219 (10)	0.0483 (3)	
H11	−0.2686	0.8599	0.5337	0.058*	
C5	−0.3968 (2)	0.94460 (18)	0.64873 (11)	0.0551 (3)	
H12	−0.5176	0.9778	0.6114	0.066*	
C4	−0.3679 (2)	0.96581 (19)	0.74939 (12)	0.0565 (3)	
H13	−0.4688	1.0137	0.7801	0.068*	
C3	−0.1888 (2)	0.91623 (19)	0.80575 (10)	0.0514 (3)	
H14	−0.1701	0.9304	0.8741	0.062*	

### Atomic displacement parameters (Å^2^)

**Table:**

	*U*^11^	*U*^22^	*U*^33^	*U*^12^	*U*^13^	*U*^23^
O1	0.0446 (5)	0.0738 (6)	0.0283 (4)	0.0085 (4)	0.0058 (3)	0.0050 (4)
O2	0.0549 (6)	0.1304 (11)	0.0483 (6)	0.0282 (6)	0.0088 (5)	0.0206 (6)
O3	0.0823 (8)	0.1176 (10)	0.0321 (5)	0.0152 (7)	0.0110 (5)	0.0155 (5)
N1	0.0432 (5)	0.0665 (7)	0.0277 (5)	0.0103 (5)	0.0052 (4)	0.0068 (4)
N2	0.0437 (5)	0.0473 (5)	0.0295 (5)	0.0021 (4)	0.0061 (4)	0.0060 (4)
N3	0.0461 (5)	0.0557 (6)	0.0329 (5)	0.0094 (4)	0.0074 (4)	0.0064 (4)
N4	0.0537 (6)	0.0657 (7)	0.0329 (5)	0.0009 (5)	0.0067 (4)	0.0096 (5)
C8	0.0646 (9)	0.0905 (11)	0.0386 (7)	0.0041 (8)	0.0004 (6)	0.0152 (7)
C7	0.0621 (8)	0.0705 (9)	0.0284 (6)	0.0037 (6)	0.0093 (5)	0.0069 (5)
C2	0.0415 (6)	0.0438 (6)	0.0360 (6)	−0.0011 (5)	0.0081 (4)	0.0035 (5)
C1	0.0398 (6)	0.0399 (6)	0.0357 (6)	0.0002 (4)	0.0072 (4)	0.0047 (4)
C9	0.0437 (6)	0.0379 (6)	0.0334 (5)	0.0025 (4)	0.0091 (4)	0.0058 (4)
C14	0.0408 (6)	0.0418 (6)	0.0338 (5)	0.0020 (4)	0.0103 (4)	0.0054 (4)
C13	0.0457 (6)	0.0422 (6)	0.0334 (6)	−0.0005 (5)	0.0083 (5)	0.0074 (4)
C12	0.0604 (7)	0.0534 (7)	0.0368 (6)	0.0060 (6)	0.0199 (5)	0.0068 (5)
C11	0.0557 (7)	0.0566 (8)	0.0524 (7)	0.0155 (6)	0.0247 (6)	0.0094 (6)
C10	0.0468 (6)	0.0493 (7)	0.0452 (6)	0.0114 (5)	0.0108 (5)	0.0109 (5)
C6	0.0466 (6)	0.0521 (7)	0.0434 (7)	0.0028 (5)	0.0024 (5)	0.0093 (5)
C5	0.0421 (6)	0.0523 (7)	0.0693 (9)	0.0062 (5)	0.0057 (6)	0.0137 (6)
C4	0.0476 (7)	0.0547 (8)	0.0704 (9)	0.0072 (6)	0.0228 (6)	0.0055 (6)
C3	0.0521 (7)	0.0577 (7)	0.0460 (7)	0.0018 (6)	0.0186 (6)	0.0019 (6)

### Geometric parameters (Å, °)

**Table:**

O1—C2	1.3672 (14)	C1—C6	1.3828 (16)
O1—C7	1.4327 (14)	C9—C14	1.3886 (15)
O2—N4	1.2153 (15)	C9—C10	1.3898 (16)
O3—N4	1.2192 (14)	C14—C13	1.3760 (15)
N1—N2	1.3295 (13)	C14—H6	0.9300
N1—C1	1.3948 (15)	C13—C12	1.3824 (17)
N1—H1	0.8600	C12—C11	1.3782 (19)
N2—N3	1.2550 (14)	C12—H8	0.9300
N3—C9	1.4165 (14)	C11—C10	1.3803 (18)
N4—C13	1.4658 (16)	C11—H9	0.9300
C8—C7	1.493 (2)	C10—H10	0.9300
C8—H1A	0.9600	C6—C5	1.3813 (19)
C8—H1B	0.9600	C6—H11	0.9300
C8—H1C	0.9600	C5—C4	1.368 (2)
C7—H2A	0.9700	C5—H12	0.9300
C7—H2B	0.9700	C4—C3	1.386 (2)
C2—C3	1.3840 (17)	C4—H13	0.9300
C2—C1	1.3996 (16)	C3—H14	0.9300
			
C2—O1—C7	117.63 (9)	C10—C9—N3	115.61 (10)
N2—N1—C1	121.19 (9)	C13—C14—C9	117.92 (11)
N2—N1—H1	119.4	C13—C14—H6	121.0
C1—N1—H1	119.4	C9—C14—H6	121.0
N3—N2—N1	112.29 (9)	C14—C13—C12	123.37 (11)
N2—N3—C9	113.57 (9)	C14—C13—N4	117.84 (10)
O2—N4—O3	122.79 (12)	C12—C13—N4	118.78 (10)
O2—N4—C13	118.69 (10)	C11—C12—C13	117.71 (11)
O3—N4—C13	118.52 (11)	C11—C12—H8	121.1
C7—C8—H1A	109.5	C13—C12—H8	121.1
C7—C8—H1B	109.5	C12—C11—C10	120.67 (11)
H1A—C8—H1B	109.5	C12—C11—H9	119.7
C7—C8—H1C	109.5	C10—C11—H9	119.7
H1A—C8—H1C	109.5	C11—C10—C9	120.44 (11)
H1B—C8—H1C	109.5	C11—C10—H10	119.8
O1—C7—C8	108.27 (11)	C9—C10—H10	119.8
O1—C7—H2A	110.0	C5—C6—C1	119.94 (12)
C8—C7—H2A	110.0	C5—C6—H11	120.0
O1—C7—H2B	110.0	C1—C6—H11	120.0
C8—C7—H2B	110.0	C4—C5—C6	120.46 (12)
H2A—C7—H2B	108.4	C4—C5—H12	119.8
O1—C2—C3	125.54 (11)	C6—C5—H12	119.8
O1—C2—C1	115.09 (10)	C5—C4—C3	120.27 (12)
C3—C2—C1	119.37 (11)	C5—C4—H13	119.9
C6—C1—N1	123.10 (11)	C3—C4—H13	119.9
C6—C1—C2	119.87 (11)	C2—C3—C4	120.09 (12)
N1—C1—C2	117.02 (10)	C2—C3—H14	120.0
C14—C9—C10	119.88 (10)	C4—C3—H14	120.0
C14—C9—N3	124.50 (10)		
			
C1—N1—N2—N3	−177.91 (10)	O2—N4—C13—C14	−2.37 (18)
N1—N2—N3—C9	−179.23 (9)	O3—N4—C13—C14	177.30 (12)
C2—O1—C7—C8	−179.64 (12)	O2—N4—C13—C12	178.57 (13)
C7—O1—C2—C3	−5.71 (19)	O3—N4—C13—C12	−1.77 (18)
C7—O1—C2—C1	175.15 (11)	C14—C13—C12—C11	−0.2 (2)
N2—N1—C1—C6	1.14 (18)	N4—C13—C12—C11	178.81 (12)
N2—N1—C1—C2	−179.72 (10)	C13—C12—C11—C10	0.1 (2)
O1—C2—C1—C6	178.70 (11)	C12—C11—C10—C9	0.1 (2)
C3—C2—C1—C6	−0.50 (18)	C14—C9—C10—C11	−0.06 (19)
O1—C2—C1—N1	−0.47 (16)	N3—C9—C10—C11	−179.95 (11)
C3—C2—C1—N1	−179.67 (11)	N1—C1—C6—C5	179.66 (12)
N2—N3—C9—C14	−2.50 (17)	C2—C1—C6—C5	0.54 (19)
N2—N3—C9—C10	177.40 (11)	C1—C6—C5—C4	−0.2 (2)
C10—C9—C14—C13	−0.07 (17)	C6—C5—C4—C3	−0.2 (2)
N3—C9—C14—C13	179.82 (10)	O1—C2—C3—C4	−179.01 (12)
C9—C14—C13—C12	0.20 (18)	C1—C2—C3—C4	0.1 (2)
C9—C14—C13—N4	−178.82 (10)	C5—C4—C3—C2	0.3 (2)

### Hydrogen-bond geometry (Å, °)

**Table:**

*D*—H···*A*	*D*—H	H···*A*	*D*···*A*	*D*—H···*A*
N1—H1···O1	0.86	2.26	2.6130 (12)	105.
C7—H2A···O3^i^	0.97	2.55	3.4595 (18)	157
C10—H10···N3^ii^	0.93	2.65	3.543 (3)	161

Symmetry codes: (i) *x*, *y*, *z*+1; (ii) −*x*+1, −*y*+1, −*z*+1.
